# Sequencing the wastewater virome: Current strategies and bioinformatic pitfalls

**DOI:** 10.1016/j.isci.2026.117596

**Published:** 2026-09-21

**Authors:** Marie De Smedt, Wim L. Cuypers, Noor Ul Hudda, Peter Delputte

**Affiliations:** 1 Laboratory for Microbiology, Parasitology and Hygiene, Department of Biomedical Sciences, University of Antwerp, Universiteitsplein 1, 2610 Antwerp, Belgium; 2 Adrem Data Lab, Department of Computer Science, University of Antwerp, Middelheimlaan 1, 2020 Antwerp, Belgium; 3 Infla-Med Centre of Excellence, Department of Biomedical Sciences, University of Antwerp, Universiteitsplein 1, 2610 Antwerp, Belgium

**Keywords:** wastewater, wastewater-based surveillance, sequencing, viruses, targeted sequencing, metagenomic sequencing, bioinformatic analysis

## Abstract

Wastewater-based surveillance (WBS) is a powerful tool for monitoring viral pathogens at the population level, complementing clinical surveillance through anonymous and unbiased insights into viral circulation. Wastewater sequencing allows to move beyond detection toward detailed characterization of circulating pathogens. This review outlines the two main sequencing strategies used in WBS: targeted and untargeted approaches. Targeted methods, including amplicon- and probe-based sequencing, provide sensitive detection and recovery of known viruses but are limited in identifying novel pathogens. Untargeted shotgun metagenomic sequencing offers a broader, unbiased view of the wastewater virome, although its performance is challenged by low human viral abundance, complex background signals, and genome assembly challenges. We also discuss key challenges complicating bioinformatic analysis, including limited human viral sequences, co-circulating viral lineages, viral dark matter, reference database limitations, large data volumes, and methodological biases, as well as emerging computational approaches aimed at improving wastewater sequencing analysis and interpretation.

## Graphical abstract

**Figure:**
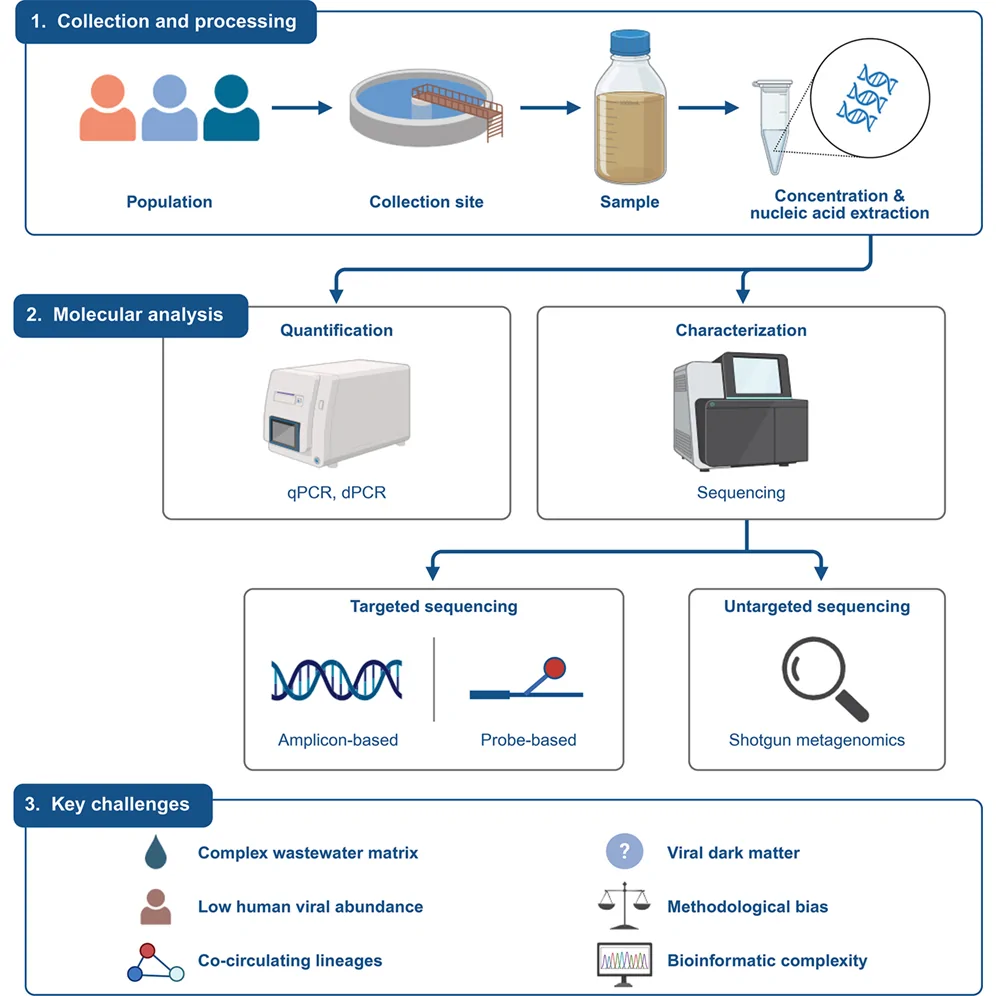

## Teaser

Public health; Environmental management

## Introduction

Wastewater-based surveillance (WBS) has emerged as a pivotal tool for monitoring infectious diseases at the population level. This method involves the analysis of wastewater for biological markers shed by humans through excretions such as feces, urine, and respiratory and nasal secretions. By tracking fluctuations in the concentration and diversity of these markers, WBS offers valuable insights into the dynamics of pathogen transmission.^1^^,^^2^ Since the excreted materials originate from a broader population, WBS provides population-wide anonymous information, circumventing biases associated with individual testing behavior or disparities in healthcare access.^2^^,^^3^

Although the concept of using wastewater to test for infectious agents dates to John Snow’s cholera research in the 19th century, modern WBS has evolved through decades of work.^4^ This was most prominently demonstrated through pathogen-specific initiatives such as environmental polio surveillance.^5^ The coronavirus disease (COVID-19) pandemic marked a turning point and demonstrated the value of wastewater as a reliable, early, and unbiased indicator of severe acute respiratory syndrome coronavirus 2 (SARS-CoV-2) circulation. In some settings, such as Belgium, SARS-CoV-2 RNA levels in wastewater even led COVID-19 incident cases up to one week.^6^^,^^7^^,^^8^^,^^9^^,^^10^ This success accelerated efforts to monitor additional viruses of public health concern, such as influenza virus (IV) and respiratory syncytial virus (RSV). WBS’ growing momentum is also illustrated by several major initiatives of the European Union (EU). In 2024, the Health Emergency Preparedness and Response Authority (HERA) launched the Global Consortium for Wastewater and Environmental Surveillance for Public Health (GLOWACON), aiming to establish an international sentinel system for wastewater surveillance.^11^ Building on this, the European Wastewater Surveillance Dashboard was launched in early 2025 and currently monitors SARS-CoV-2, IV, and RSV across 13 European countries.^12^ In December 2025, the European Center for Disease Prevention and Control (ECDC) further complemented these efforts by establishing a framework to integrate WBS into EU infectious disease surveillance, positioning it as a routine component of public health monitoring in line with legislative requirements such as the Urban Wastewater Treatment Directive.^13^

As illustrated in Figure 1, wastewater from an upstream population is commonly centralized in wastewater treatment plants (WWTPs) where samples are mainly collected at the inlet (influent wastewater), although outlet samples (effluent wastewater) are also used. Wastewater can also be sampled from sewage networks of large buildings, such as university campuses and hospitals, or from non-sewered systems, such as rivers and canals.^14^^,^^15^^,^^16^ Samples are then processed to concentrate the target—e.g., viral particles or bacterial cells—and extract genetic material, after which target quantification can be performed using molecular assays such as quantitative or digital polymerase chain reaction (q/dPCR).^17^^,^^18^ While targeted quantitative molecular detection remains central for rapid and cost-effective determination of viral load trends for specific pathogens, another application in WBS that is increasingly applied is genomic sequencing. Sequencing allows WBS to move beyond detection toward detailed characterization and a deeper understanding of the circulating pathogens.^19^^,^^20^ Targeted sequencing approaches allow the identification of known viruses or variants by prior enrichment, while metagenomic or untargeted sequencing provides a broader perspective by capturing the wide diversity of viral genomes present in wastewater without assumptions. This enables the detection of unexpected, emerging, or co-circulating viruses without prior knowledge of their presence, and as such is also a major asset for pandemic preparedness.^10^^,^^19^^,^^21^^,^^22^

**Figure 1:**
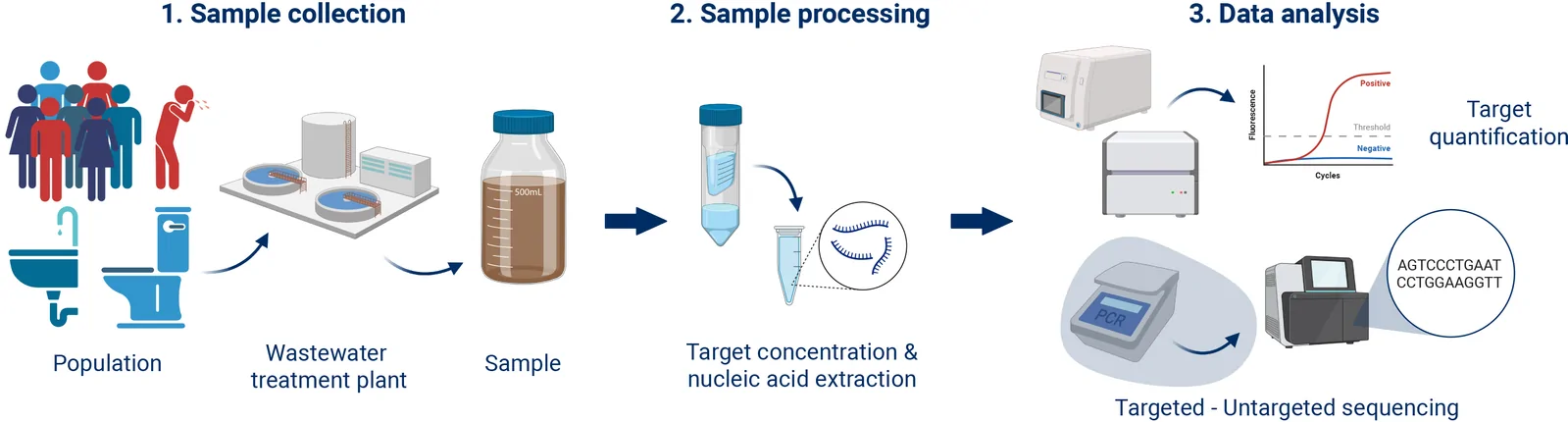
General workflow of wastewater-based surveillance and wastewater sequencing Wastewater is commonly centralized in wastewater treatment plants, where samples are collected. Samples are then processed to concentrate and extract target genetic material. Target quantification can be performed using molecular assays such as quantitative or digital polymerase chain reaction and target characterization can be performed using targeted or untargeted sequencing approaches. Figure created with BioRender.

The ability of wastewater sequencing and WBS to provide population-wide, non-invasive, and privacy-friendly insights makes it a powerful complement to traditional clinical surveillance and sequencing.^9^^,^^23^ However, despite rapid progress, this surveillance strategy’s full potential for public health and pandemic preparedness purposes remains under development. Significant challenges remain, particularly with respect to the complexity of bioinformatics for processing and interpreting sequencing data. In this review, we describe the current state of knowledge on wastewater sequencing by first discussing targeted and untargeted sequencing approaches. We then focus on the challenges related to the downstream analysis, where advanced bioinformatics pipelines are needed to extract meaningful data from the highly heterogeneous and data-rich outputs. Consideration of these critical aspects in future efforts is needed to improve our understanding of how wastewater sequencing can reach its full potential as a cornerstone for public health surveillance.

## Current wastewater sequencing strategies

For virus-focused wastewater sequencing, viral particles are usually first concentrated from the wastewater sample. The genetic material is released only after lysis, yielding pure DNA or RNA, or a mixture of DNA and RNA, depending on the method used. These may either undergo an additional concentration step or be prepared directly for downstream analysis. Diverse sequencing workflows exist, especially regarding whether prior target enrichment is performed (Figure 2). Targeted sequencing relies on such prior target-specific enrichment, while untargeted sequencing avoids selective enrichment for predefined targets. The flow usually consists of nucleic acid fragmentation, followed by library preparation and sequencing. For RNA targets, RNA is first reverse transcribed into complementary DNA (cDNA) to create a template compatible with most sequencing workflows, which often require DNA. DNA is then fragmented into shorter pieces suitable for library preparation, where sequencing adapters are ligated to the fragment ends, enabling attachment to the sequencing platform and subsequent amplification where applicable (e.g., in PCR-based library preparation protocols). The prepared library is then sequenced to generate reads representing the genetic material in the sample.

**Figure 2:**
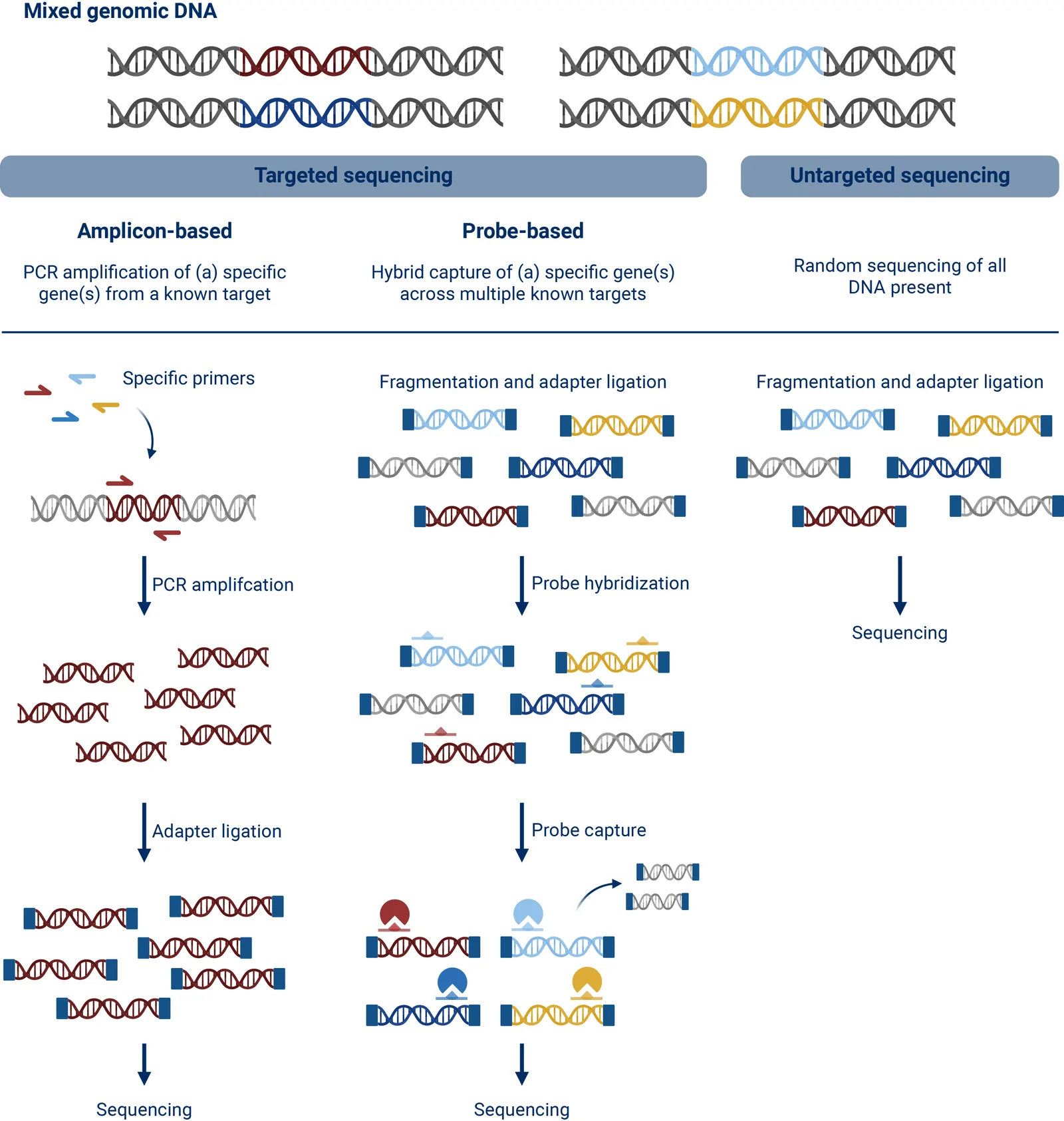
Targeted and untargeted sequencing strategies for wastewater-based surveillance Wastewater contains mixed genetic material from diverse organisms. Amplicon-based sequencing selectively amplifies specific genes from a known target organism using PCR primers. Probe-based or hybrid-capture sequencing enriches predefined genes across multiple known targets through probe hybridization and subsequent capture. Untargeted shotgun metagenomic sequencing allows a target-independent approach by sequencing all DNA present. Figure created with BioRender.

General features of these targeted- and untargeted sequencing strategies, including their primary strengths and limitations and best use cases, are summarized in Table 1 based on reports across multiple studies.^21^^,^^22^^,^^24^^,^^25^^,^^26^^,^^27^^,^^28^^,^^29^^,^^30^^,^^31^

**Table 1:** Key aspects of targeted (amplicon- and probe-based) and untargeted shotgun metagenomic sequencing

	Amplicon-based targeted sequencing	Probe-based targeted sequencing	Untargeted shotgun metagenomic sequencing
Enrichment strategy	(tiled-) PCR amplification using virus-specific primers	hybridization to oligonucleotide probes followed by capture	no predefined target-specific enrichment; direct sequencing
Step in sequencing workflow	before adapter ligation, no need for further fragmentation	after fragmentation and adapter ligation	direct sequencing after fragmentation and adapter ligation
Prior knowledge required	high; knowledge of target virus for primer design	moderate; knowledge of viral families/species for probe design	none
Virus detected per assay	typically one per primer scheme	one to thousands (using probe panels)	all present (theoretically)
Detection of novel/emerging viruses	limited; novel variants may be missed if primer sites mutate	moderate to high; probes can bind conserved regions even with divergence	moderate to high; target-independent detection, although constrained by background interference
Sensitivity for low-abundance human viruses	very high for known targets	high	low due to background interference
Tolerance to sequence mismatches	low; primer mismatches can cause amplicon dropout	high; probes tolerate mismatches and can bind conserved regions	high
Impact of wastewater background	strongly reduced by PCR enrichment	strongly reduced by probe capture	strongly affected; bacteriophages and plant viruses dominate reads
Need for frequent updates	high; primers must be redesigned as viruses mutate	moderate; probe panels need updates	none
Bioinformatic complexity	moderate	moderate to high	high
Primary strengths	high sensitivity, well-validated protocols	broad viral detection, detection of divergent or emerging viruses	unbiased detection, discovery of novel pathogens, comprehensive virome profiling
Primary limitations	limited to known viruses, vulnerable to primer mismatches, single-virus focus, less adaptable to emerging targets due to the need for primer redesign and optimization	variable panel performance, panel updates require new probe design and procurement	largely affected by wastewater background, low sensitivity for low-abundance human viruses, data-intensive
Best use case	high-resolution genomic surveillance of a known virus	broad surveillance of several known and emerging viruses simultaneously	unbiased pathogen surveillance and discovery
Running costs and operational burden once protocol is optimized	low; fast turnaround time, low to medium sample preparation costs, low computational demand	medium; moderate turnaround time, medium to high sample preparation costs, medium computational demand	high; slow turnaround time, high sample preparation costs, high computational demand

### Targeted sequencing: Amplicon- and probe-based approaches

Wastewater samples are highly heterogeneous mixtures that contain fragmented genetic material from numerous human, animal, and environmental sources. This complexity particularly poses challenges when sequencing viruses, since they are often present only in low quantities, with signals diluted by the complex background.^22^^,^^32^ Additionally, a wide range of viruses may be present, some infecting humans while others infect plants, insects, algae, or bacteria.^33^ To cope with these challenges and generally improve recovery of these low-abundance targets, nucleic acids of interest can first be enriched, a process called targeted sequencing. The two main strategies are (1) amplicon-based sequencing and (2) probe-based or hybrid-capture (hybridization-capture) sequencing. Both strategies aim to enhance sensitivity by reducing background noise from non-target genetic material, yet differences in their underlying principles determine their suitability for detecting known or novel and emerging viruses. It is worth noting that, regardless of the approach, these enrichment workflows require longer processing times compared to rapid quantitative assays such as dPCR (several days compared with hours), often limiting rapid responses to emerging diseases when needed.^34^

#### Amplicon-based sequencing

Amplicon-based sequencing relies on PCR primers designed to amplify genomic regions of interest. This results in multiple amplicons, removing the need for fragmentation and allowing direct adapter ligation (Figure 2). The primers can be applied using tiled primer schemes to generate overlapping amplicons that cover the entire genome, enabling whole-genome sequencing.^22^^,^^24^^,^^25^ Based on the primer scheme, amplicons of different lengths can be generated. Using a primer scheme with shorter amplicons makes use of more primers, thereby increasing the primer-pool complexity and raising the likelihood of primer-primer interactions, reducing genome coverage.^35^ The resulting abundance of small, fragmented reads—combined with potential gaps in coverage—may make it more difficult to confidently reconstruct full viral genomes during assembly. Although this technique is reliable, sensitive for known targets, and relatively cost-effective with short sample-preparation times, its reliance on predefined primer sets limits the genomic variability that can be captured, thereby reducing its ability to detect unexpected or novel variants.^22^^,^^26^^,^^27^ As viruses accumulate mutations that allow them to escape PCR and subsequent sequencing, frequent updating and redesigning of the primers is also necessary.^26^^,^^28^ Moreover, primer-binding site mismatches can lead to reduced or failed amplification of certain amplicons or entire genomic regions. This can potentially cause variants of epidemiological importance to be missed.^21^

Universal tiled amplicon-based sequencing protocols exist, such as the ARTIC Network protocols for SARS-CoV-2, measles virus, Ebola virus, and mpox virus.^36^ Unlike variant- or mutation-specific assays, which are designed to target specific variants or mutations, this approach supports the detection of both known and novel variants, including extremely low-abundance viruses that would otherwise require multiple rounds of amplification.^27^ Such universal protocols are well validated, optimized for specific viruses, cost-effective, and scalable, making them easier and faster to implement than developing primer schemes from scratch. In addition, open-source tools such as PrimalScheme provide researchers with accessible platforms to design custom schemes for diverse viruses (preprint).^37^ It is, however, to be noted that primer pools from such protocols can still experience amplicon dropout or reduced coverage in regions where the template DNA is fragmented or primer-binding sites have mutated, and are inherently limited to only one virus per primer scheme.^19^^,^^27^ Targeted amplicon-based sequencing has been used successfully in wastewater for numerous pathogens, providing detailed insights into viral presence. Recent examples and their key findings are summarized in Table 2.

**Table 2:** Summary of the principal manuscripts included this review, highlighting key findings, methodological approaches, and reported challenges according to sequencing strategy

Study	Target(s)	Platform	Pipeline/database	Read count and depth/coverage (where reported)	Key outcome	Main limitations/comments
**Amplicon-based targeted sequencing**						

John et al.^38^	influenza A virus (IAV) H1N1/H3N2	ONT Nanopore	Dorado; V-pipe (samtools bwa, LoFreq, VILOCA); lollipop; GISAID database	up to ∼10^4^ reads per amplicon, outliers up to 10^5^; up to ∼10^4^ position-wise read depth (visual estimates from plots)	wastewater H1N1 abundance correlated with clinical data; clinically relevant mutations in vaccine- and drug-target sites detected	primer dropout reduces coverage; new mutations require primer updates; non-viral-load factors (matrix/ methodology/…) influence sequence efficiency
Lee et al.^39^	pan-IAV	ONT Nanopore (whole-genome sequencing)	Guppy; Centrifuge; minimap2; samtools & BCFtools; BLAST+; Nextclade; IAV reference genomes	total reads per sample ranged from 65 to 1440; average percentage coverage per segment ranged from 25.7% to 91.3%	dynamic IAV signal detected with no clear correlation to clinical data; meta whole-genome sequences showed homology to human and avian IAV strains (diverse subtypes and lineages)	reads biased to smaller genome segments; no sample achieved complete genome coverage; matrix complexity affects sequencing performance; post-PCR contamination carryover impairs flow cell performance
Veneri et al.^40^	human adenovirus (hAdV)	ONT Nanopore	Guppy (HAC); minimap2; custom hAdV genome database	detection was confirmed only for genotypes with ≥ 30*×* read depth	multiple co-circulating and low-abundance genotypes detected; enhanced resolution compared to Sanger sequencing	reliance on a single target region causes genotyping ambiguities; practical constraints (time/cost/resources) necessitated sample pooling
Giesbrecht et al.^41^	human papilloma virus (HPV)	Illumina MiSeq	MiSeq Control Software; Trimmomatic; PEAR; NCBI BLAST+; custom Papillomavirus Episteme-derived database	average of 237,531 reads per sample (*n* = 73), of which in total across all samples 11,148,715 aligned to an HPV-type	24 distinct HPV types detected, including cutaneous and mucosal types of high, low, and unknown risk	primer-target mismatches cause amplification inefficiency

**Probe-based targeted sequencing**						

Karatas et al.^42^	TWIST Comprehensive Viral Panel	AVITI	EsViritu; Virus Pathogen Database	> 3 million total reads mapped to viruses (*n* = 6; visual estimates from plots); reads were mapped with 90% percentage coverage criteria	2294 viral genomes or segments recovered, spanning 51 animal- and 135 human-associated viral species	detection is biased toward known or closely related viruses (probe efficiency limited to sequences with > 80% nt similarity); limited assessment of viral population complexity (analysis focused on consensus genomes)
Tisza et al.^20^	TWIST Comprehensive Viral Panel	Illumina NovaSeq 6000	EsViritu; Virus Pathogen Database	median 28.5 genomes/segments covered over > 90% of their length, additional 41 covered over > 50% of their length (percentage coverage; *n* = 363)	broad community viral diversity: 465 virus strains detected with reads correlating with clinical data for some	insufficient genome coverage for reliable allele/variant frequency analysis; low viral shedding limits coverage; Improved enrichment and increased depth are required to enhance coverage and variant analysis
Zulli et al.^34^	Illumina Viral Surveillance Panel	Illumina NovaSeq 6000	Chan-Zuckerberg Infectious Disease mNGS Illumina; minimap2; SPADES; bowtie2; NCBI NT/NR databases	∼40 million reads per sample; 3%–90% percentage coverage, 5- to 20-fold read depth	33 respiratory panel viruses detected, enabling seasonal monitoring of outbreak timing and severity (including viruses with limited clinical surveillance)	practical constraints necessitated sample pooling; varying coverage breadth and depth among targets (influenced by viral concentration/outbreak activity); non-customized bioinformatics workflow limits analysis performance; matrix complexity affects sequencing performance
Holland et al.^43^	Illumina Respiratory Virus Oligo Panel v2	Illumina	Bbtools; BWA; samtools; custom database	median 6.6 × 10^6^ reads per sample; only samples with read depth > 10 were reported	hAdV surge in university-campus wastewater prompting targeted clinical testing that confirmed outbreak; shared SNPs found, linking community transmission to outbreak	differences in probe-specificity between subtypes may skew read counts quantitatively

**Shotgun untargeted metagenomic sequencing**						

Worp et al.^33^	–	Illumina MiSeq	Snakemake; FastP; CD-HIT; BWA-MEM; samtools; metaSPAdes; DIAMOND; geNomad; NCBI nr database	median 9.8 × 10^5^ reads per sample; each assembled contig had ≥ 3 read depth	122 viral families detected; bacteriophages and plant viruses most dominantly, viral families linked to human disease also detected in high abundances	high number of reads unassigned to known viral taxa; low genomic resolution in critical regions due to partial genome recovery limits assignment to known taxa
Rushford et al.^44^ (preprint)	–	Illumina NovaSeq 6000/NovaseqX	Snakemake; BBMap; NCBI STAT (prefiltering); SPAdes; BLAST (NCBI nt); minimap2; samtools; NCBI reference databases	average 1.1 billion reads per sample; e.g., percentage coverage ranged from 31%–100% for ICV	common enteric-, less abundant respiratory-, and unexpected viruses detected (avian IV H5N1, hepatitis A surge, spike in ICV)	dominance of ribosomal RNA (rRNA) reads dilute target signals; rRNA depletion risks loss of target reads; limited classification indicates need for dedicated wastewater-associated database

Manuscripts were selected based on publication within the last three years to ensure an overview of recent work and evidence.

#### Probe-based sequencing

Besides PCR-amplification, enrichment can also be achieved using oligonucleotide probes that hybridize to target genetic material, a method known as probe-based or hybrid-capture sequencing. This approach is applied after fragmentation and adapter ligation to the DNA template (Figure 2). Following hybridization, a capture step separates the targets from background material.^29^ Like amplicon-based sequencing, this approach enhances the sensitivity and can recover larger genomic regions, including whole genomes.^22^ However, whereas amplicon-based sequencing typically targets only a single virus per assay, probe-based sequencing can simultaneously target a greater diversity of viruses by using probe panels. These allow the detection of tens to hundreds of viruses in the same sample, often making it more economical on a per-target basis.^30^ The panels can enrich entire viral families or species and can substantially increase human-virus coverage by specifically targeting human viruses.^29^ Several commercial panels exist, including the Illumina Respiratory Virus Oligos Panel (RVOP),^22^^,^^43^^,^^45^ Respiratory Pathogen ID/AMR Panel (RPIP),^22^ Twist Comprehensive Viral Research Panel,^46^ and QIAseq xHYB Adventitious Agent Panel.^26^^,^^47^ Among these, the Twist panel is mainly used, capable of detecting over 3,000 human and animal viruses, as exemplified by Tisza et al. (2023) who observed over 450 distinct pathogenic viruses from 28 viral families, and Karatas et al. (2025) who detected more than 160 different viral species infecting humans and animals.^20^^,^^42^^,^^46^

One of the key advantages of probe-based sequencing is that probes may hybridize even when there is partial sequence divergence and can capture short conserved sequence motifs within larger genome fragments. This enables the detection of novel or emerging viruses, even when they are not part of the panel targets.^26^^,^^28^^,^^29^ Despite significant progress in this field, a universally standardized pipeline has yet to be established, and available probe panels display considerable variation in sensitivity, enrichment capacity, and overall performance.^21^^,^^26^ Jiang et al. (2024) have demonstrated that the detection of low-abundance viral species can differ between panels, even if they are within the same viral genus. Additionally, they showed that seasonal changes in wastewater composition and concentration and nucleic acid extraction protocols also likely influence probe-capture success.^29^ Successful recent applications of probe-based sequencing are summarized in Table 2.

### Untargeted, shotgun metagenomic sequencing

Enrichment-based targeted sequencing methods provide high sensitivity, yet they are mostly restricted to known pathogens. In contrast, untargeted shotgun metagenomic sequencing offers a target-independent strategy for the detection of viral presence and newly emerging pathogens, improving understanding of viral evolution, pathogenesis, and transmission.^19^^,^^22^^,^^28^ Without the need for prior knowledge and specific primer or probe design, it can capture genetic material from all domains of life present in wastewater, enabling rapid and multi-species monitoring.^19^^,^^22^ Its effectiveness, however, is limited by the dominance of non-virus material and the natural composition of the sewage virome. Wastewater is dominated by genetic material mostly from bacteria and other prokaryotes.^48^^,^^49^ Even when viral particle-enrichment methods, such as removal of solids and bacterial cells, are applied, Child et al. (2023) reported fewer than 0·6% of shotgun metagenomic reads could be assigned to viruses.^22^ Moreover, the detectable sewage virome mostly consists of bacteriophages and plant viruses, which significantly reduce the relative signal originating from human viruses.^33^^,^^50^^,^^51^ The high genetic diversity of viral groups—driven by frequent mutation, recombination, and reassortment—additionally complicates the later assembly and annotation of complete genomes from these numerous sequencing reads, particularly when using a short-read platform. Long-read sequencing technologies can help mitigate these challenges by generating longer sequences that facilitate genome reconstruction.^28^ A comparative study of untargeted metagenomic sequencing, amplicon-based targeted sequencing, and probe-based targeted sequencing showed that untargeted sequencing may fail to achieve sufficient genome coverage for reliable genomic surveillance. In contrast, probe-based enrichment was considered a more effective approach, and tiled-amplicon-based sequencing provided the most complete genome coverage, although for individual viruses.^22^ Nevertheless, shotgun metagenomics has recently been applied successfully, of which a selection of studies is summarized in Table 2.

### Sequencing platforms

Regardless of the sequencing strategy, users can choose between short-read or long-read platforms. These platforms generate reads of different lengths, each offering distinct advantages, but also with trade-offs between coverage, read depth, and error rate that ultimately influence overall data quality and viral variant detection. Platform selection is therefore guided by the actual surveillance goals—which may include routine monitoring, broad pathogen discovery, detailed viral characterization, or a combinatorial approach—as well as the required sequencing resolution, turnaround time, and costs. One of the most widely used short-read platforms, namely Illumina, relies on sequencing-by-synthesis, where fluorescently labeled reversible terminator nucleotides are added one at a time, allowing base-by-base identification (Figure 3A). This approach provides high read depth and high base quality at a relatively low sequencing cost per sample when multiplexing multiple samples, although it has limited ability to resolve linked mutations within individual genomes. In contrast, nanopore sequencing, as marketed by Oxford Nanopore Technologies (ONT), is a third-generation platform that infers nucleotide identity from changes in electric current as nucleic acids pass through a protein nanopore (Figure 3B). The ONT platforms enable the generation of long reads that can span entire amplicons or even complete viral genomes, improving genome assembly. This is particularly advantageous in wastewater contexts, given the complex and heterogeneous nature of the genetic material present. Despite these advantages, ONT long-read sequencing has relatively higher error rates and can be more costly depending on throughput and application, complicating accurate base calling and often requiring error-correction strategies, although this continues to improve with ongoing advances in ONT chemistry and base calling models.^28^^,^^52^

**Figure 3:**
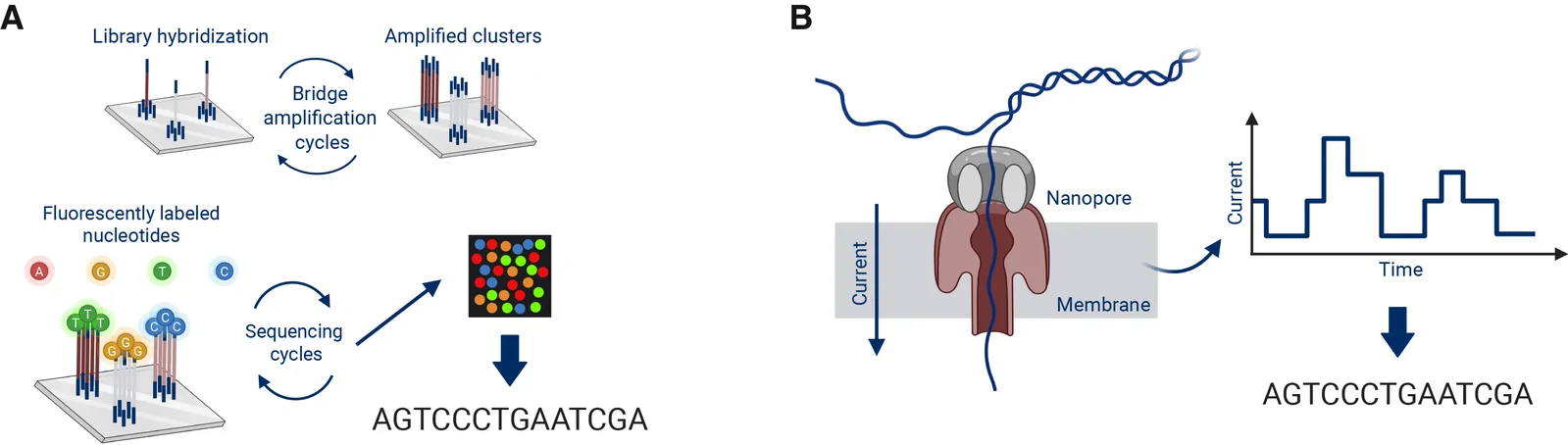
Overview of Illumina and Oxford Nanopore Technologies sequencing platforms (A) Illumina relies on a sequencing-by-synthesis workflow, where DNA is immobilized, amplified, and sequenced through repetitive incorporation of fluorescently labeled reversible terminator nucleotides to generate short, high-accuracy reads. For the (B) ONT platform, single nucleic acid molecules pass through a protein nanopore and nucleotide sequences are inferred from characteristic changes in ionic current over time, enabling long-read sequencing. Figure created with BioRender.

## Downstream analytical challenges in viral wastewater sequencing

To achieve reliable pathogen detection and generate epidemiologically meaningful insights, it will be crucial to integrate both targeted and untargeted sequencing approaches, making use of multiple sequencing platforms.^19^^,^^20^ Combining these strategies will require parallel library preparation, separate downstream analyses, and careful harmonization of the results.^52^ However, at this moment, wastewater sequencing as a whole continues to face substantial analytical and interpretative challenges.

Both targeted and untargeted sequencing approaches generate large data volumes, often in the megabyte-to-gigabyte range per sample.^27^^,^^32^ To extract relevant information from these datasets, one of the most important challenges is the inherent low abundance of human viral nucleic acids relative to non-human viral, bacterial, and environmental genetic material. Highly abundant background nucleic acids dominate sequencing reads, thereby overshadowing the human viral signal and complicating downstream bioinformatic analysis.^33^^,^^50^^,^^51^ As discussed, enrichment-based sequencing strategies can partially overcome this challenge by selectively amplifying or capturing the virus of interest, though they are mostly constrained to known viral targets and may fail to detect novel or divergent variants, which may be detected by shotgun metagenomics.^19^^,^^22^^,^^26^^,^^27^ Furthermore, in PCR-based workflows, stochastic effects, polymerase errors, and occasional template switching and amplification bias can introduce artifacts into sequencing results, misrepresenting the actual viral diversity and its relative abundance.^53^ Another option to increase the limited human viral fraction is to consider the upstream methodological choices. Sampling strategy, among others, can introduce an initial source of bias, as the type and timing of sample collection influence how accurately wastewater reflects true viral concentrations. Two commonly used strategies are grab samples, which capture wastewater at a single time point, and composite samples, which consist of pooled subsamples typically collected over a 24-h period, often at fixed time intervals (time-proportional) or in proportion to the wastewater flow (flow-proportional). Composite samples provide a more representative view of viral variation over time, with flow-proportional sampling recommended for the most reliable viral load estimation.^23^^,^^54^ Additional viral gains can also be achieved by optimizing concentration and preprocessing steps. Viral particles are distributed across both the liquid and solid fractions of wastewater, and their partitioning depends on virus-specific properties such as envelope status, size, and surface characteristics. Consequently, the use of a concentration method that targets the wrong fraction can substantially reduce recovery of the target virus, as has been demonstrated for both quantitative molecular assays and sequencing-based approaches.^26^^,^^55^^,^^56^^,^^57^^,^^58^^,^^59^^,^^60^ Studies focusing on sequencing have shown that different concentration methods vary in their recovery efficiencies, influencing sequencing sensitivity, genome coverage, and the composition of the recovered virome.^57^^,^^58^ As a result, the choice of concentration strategy is a major source of methodological bias and can reshape the perceived viral community structure. For example, Segelhurst et al. (2025) demonstrated that different concentration approaches enriched distinct RNA viromes from the same wastewater samples, likely because of differences in virus partitioning between and method-specific recovery mechanisms.^58^ The impact of preprocessing is also particularly important for PCR-based sequencing workflows, where concentration procedures may also co-concentrate PCR inhibitors. Variability in inhibitor carryover can affect amplification efficiency, sequencing quality, and the detection of low-abundance pathogens, making effective inhibitor removal a critical consideration during method selection and optimization.^59^^,^^60^ Beyond concentration, enrichment and depletion strategies can further improve sequencing performance by increasing the virus-to-background ratio.^26^ These approaches include target enrichment through probe-based capture as well as the removal of non-target nucleic acids to reduce host and environmental background signals. Tailoring preprocessing protocols to the specific properties of the virus of interest can therefore enhance recovery of the human viral signal, providing an initial reduction of background noise and improving downstream analytical sensitivity. Overall, an integrated approach spanning sampling design, preprocessing workflows, and sequencing strategy will be required to minimize potential biases.

Beyond their limited abundance in wastewater, human viruses also present an additional challenge due to the simultaneous circulation of viral lineages and the emergence of recombinant forms. This diversity complicates lineage deconvolution—the process of separating and estimating the relevant abundance of viral lineages to track variant dynamics in populations.^22^^,^^28^ Recent advances, particularly those developed during the COVID-19 pandemic, have improved the feasibility of such analyses. Notably, Freyja, which estimates the relative abundance of co-circulating lineages using lineage-defining single-nucleotide variant barcodes and depth-weighted regression models, and CoOccurrence adJusted Analysis and Calling (COJAC), which detects the co-occurrence of multiple variant-specific signature mutations on read pairs to provide high-confidence variant identification, have demonstrated the potential to increase resolution in complex viral mixtures such as wastewater.^61^^,^^62^

Consequently, extracting information on human viral presence from sequencing datasets is already a significant challenge, requiring substantial computational resources, time, and specialized analytical skills or expertise.^27^^,^^32^ Several analytical parameters further influence data interpretation and comparability. These include (1) the similarity or match cutoff, which defines the minimum sequence similarity required for a read to be considered a valid match to a reference and directly affects the balance between false positives and false negatives; (2) detection thresholds, which determine the minimum evidence required to confidently call a virus present in a sample, including minimum read counts or read depth; and (3) the use of negative controls or blanks to account for background noise, where the choice of blank influences what is considered a signal or not.^26^^,^^32^ More stringent criteria generally reduce false positives but increase the likelihood of missed detections, whereas more permissive thresholds improve sensitivity at the cost of false discoveries. For targeted sequencing strategies, downstream analytical complexity is partially eased by prior enrichment of the virus of interest, although a certain level of expertise is still required to ensure accurate variant identification. In contrast, shotgun metagenomic sequencing may be considered more challenging, as all DNA present is sequenced, generating large datasets that require advanced bioinformatic tools.^63^^,^^64^

Currently, as evident from Table 2, no standardized or *one-click* analytical workflow exists that enables fully automated, user-independent analysis and interpretation of sequencing data for either sequencing strategy. One typically relies on either assembly-based or read-based approaches for viral identification. Assembly-based methods first reconstruct sequencing reads into longer contigs before classification. Because contigs contain more genomic context than individual reads, they can enable more reliable taxonomic assignments, support genome reconstruction and phylogenetic analyses, and improve the detection of divergent viruses. These advantages make assembly-based approaches particularly valuable for tracking viral diversity and evolution. However, successful assembly requires sufficient sequencing depth and genome coverage, meaning that low-abundance viruses may not be recovered. Furthermore, assembly can be computationally intensive and may introduce biases associated with assembly quality and parameter selection.^20^^,^^65^^,^^66^^,^^67^ In contrast, read-based approaches classify individual sequencing reads directly and can therefore detect viral signals even when genome coverage is insufficient for assembly. This sensitivity makes them attractive for routine surveillance and the early detection of emerging pathogens at low abundance. However, classifications based on short reads generally provide less genomic context and are more susceptible to ambiguous assignments, false positives, and database-related biases, particularly at lower taxonomic levels.^66^^,^^67^ The two approaches should therefore be viewed as complementary rather than competing strategies. Read-based analyses can provide sensitive detection of low-abundance viral signals, while assembly-based analyses enable more confident taxonomic classification and downstream genomic characterization. This complementary framework is reflected in tools such as hecatomb, which integrates both read-level and contig-level viral identification to leverage the strength of each approach.^68^ With advances in artificial intelligence, machine-learning-based tools such as DeepVirFinder, VIBRANT, and the recent Metagenomics-Toolkit have also been developed for the analysis of metagenomic reads.^69^^,^^70^^,^^71^ These approaches learn informative features from input sequence data and predict viral identity as output, demonstrating improved accuracy in identifying viral sequences. In addition, tools such as branchwater and Logan-Search enable more rapid screening of large genomic databases using sketch-based methods (the use of compact summaries of the detected sequences instead of full sequences) and facilitate genome reconstruction, respectively (preprints).^32^^,^^72^^,^^73^

Another significant obstacle in shotgun metagenomic sequencing is the extensive presence of *viral dark matter*, the large fraction of recovered sequences that does not align with any reference sequence. They either cannot be assigned to known viral taxa or are distantly related to species described in public sequence databases. Its presence complicates the characterization of the wastewater virome and limits the extraction of biologically and epidemiologically meaningful information, thereby increasing uncertainty in downstream interpretation, risk assessment, and public health relevance. Although it poses a significant analytical obstacle, an extensive amount of potentially relevant information also resides within this unclassified fraction.^63^^,^^64^ Employing these sequences provides the opportunity not only to discover novel viral lineages but also to construct more complete and representative reference databases, as the choice of database strongly influences the results of wastewater sequencing. Curated databases reduce the risk of misclassification but often lack representative wastewater-specific viral diversity, whereas larger, less curated databases increase the likelihood of detecting environmentally relevant viruses, at the risk of taxonomic misclassification.^26^^,^^32^ This highlights the growing importance of including sequences recovered through culture-independent approaches, such as metagenome-derived viral genomes or uncultivated virus genomes (UViGs), to help fill these gaps and improve downstream analyses.^74^ Standardized reporting and protocols and rigorous quality control of such sequences are, however, essential to ensure their reliability and value when integrated into public resources.^75^ Databases like integrated microbial genomes/virus (IMG/VR) and viral integrated resource across ecosystems (VIRE), or those discussed by Ritsch et al. (2023), now incorporate large numbers of UViGs from diverse environments, allowing more accurate virome characterization, enhancing our understanding of the global virome and supporting virus taxonomy refinements.^74^^,^^76^^,^^77^ Building and maintaining such databases—incorporating all viral sequences recovered, including those who are unclassified—may ultimately help to determine more accurately whether newly detected sequences are genuinely novel or whether an apparent absence in a sample reflects a true negative rather than a limitation of existing references. Without representative reference databases, analyses may suffer from substantial representation bias, limiting both the interpretability and comparability of wastewater virome studies.

Finally, viral sequencing from wastewater offers the exciting opportunity to infer virus-host associations, although realizing this potential remains challenging. Direct evidence- or alignment-based approaches infer hosts from molecular signatures of virus-host coevolution and can provide high-confidence assignments, but their applicability is limited by incomplete reference databases and substantial computational requirements, especially for metagenomic datasets.^78^ Alignment-free methods, such as the k-mer-based tools phage–host interaction search tool (PHIST) and Who Is the Host? (WIsH), compare sequence composition between viruses and potential hosts and are generally faster, although often at the cost of reduced accuracy.^78^^,^^79^ CRISPR-based approaches exploit matches between viral sequences and host-encoded CRISPR spacers, but their utility is restricted as many hosts lack CRISPR systems and only a subset share detectable spacer matches.^78^ More recently, integrative and machine-learning-based frameworks, including integrated Phage-Host Prediction (iPHoP), Computational metHod for accuratE pRediction of virus–pRokarYotic interactions (CHERRY), and virus-host interactions predictorVHIP, have improved host prediction performance by combining multiple sources of evidence to infer virus-host associations.^80^^,^^81^^,^^82^ It should be noted, however, that these methods were developed primarily for viruses infecting prokaryotic or microbial hosts. In contrast, host prediction methods for human-associated and other eukaryotic viruses remain underdeveloped. Further research in this area could improve future zoonotic disease surveillance efforts by enabling the identification of viruses with zoonotic spillover potential.^83^ Despite these advances, host prediction remains hindered by reference database bias, high computational demands, and limited generalizability to poorly characterized viruses and hosts. Nevertheless, host information can provide ecological context, as for example Worp et al. (2022) have shown by using the Virus-Host Database to classify viral families according to host range.^33^^,^^84^ This database integrates virus-host associations from resources such as NCBI RefSeq, GenBank, UniProt, and ViralZone, providing a valuable foundation for ecological analyses and host prediction.^84^

## Complementary roles of wastewater and clinical sequencing

For some, discordance between wastewater sequencing results and clinical sequencing results is viewed as a limitation, although it more accurately reflects the complementary nature of these approaches. Rather than indicating shortcomings, such discordance highlights the value in each method: wastewater sequencing captures viral signals from an entire population, including asymptomatic or untested individuals, whereas clinical sequencing provides patient-level data that can be linked to clinical outcomes.^9^^,^^23^ This complementarity is evident across multiple examples. WBS and wastewater sequencing have repeatedly demonstrated their ability to detect and track pathogens independent of clinical case reporting, such as accurately reconstructing the spread of the SARS-CoV-2 Omicron variant across England,^85^ and identifying wild-type poliovirus type 1 in Germany in November 2025—genetically linked to a sample from Afghanistan—despite the absence of clinical cases.^86^ Similarly, Holland et al. (2024) showed how wastewater systems can serve as an early warning tool in outbreak-prone settings. They detected a surge in human adenovirus in university-campus wastewater, which prompted targeted clinical testing that confirmed the outbreak. Shared single-nucleotide polymorphisms across these wastewater and clinical samples were found, linking community transmission to the outbreak.^43^ At the same time, strong concordance has also been observed between wastewater and clinical sequencing strategies, particularly for SARS-CoV-2, with both approaches further capable of identifying unique mutations, underscoring their complementarity.^27^^,^^87^^,^^88^ Together, these observations indicate that some discordance is expected, but informative, reinforcing that wastewater and clinical sequencing provide synergistic insights that enhance population-level surveillance and early detection efforts.

## Research gaps and future directions

Wastewater sequencing has become a critical component of WBS, offering deeper insights into viral diversity, transmission dynamics, and evolution at the population level. When combined with clinical surveillance strategies, these systems can reveal the true societal burden of infectious agents and fill gaps that arise when infections are asymptomatic or underreported or when clinical testing declines. While substantial progress has been made, this review shows that several methodological and analytical challenges must be addressed before its full potential can be realized.

A key priority is the standardization of sequencing approaches across laboratories. For targeted approaches such as amplicon- and probe-based sequencing, greater protocol sharing and development of best-practice guidelines are needed to improve reproducibility and comparability between studies and surveillance programs. Establishing consensus approaches for assay design, validation, quality control, and data reporting would facilitate more reliable interpretation of sequencing results across regions. In addition, no single strategy can currently address all surveillance objectives. Targeted methods provide the sensitivity and genome coverage required for monitoring known pathogens, whereas shotgun metagenomics remains essential for broader pathogen discovery and advancing our understanding of the wastewater virome. Future work should therefore focus on optimizing integration of these complementary approaches to specific surveillance needs rather than pursuing a universal *one-size-fits-all* solution. For shotgun metagenomic sequencing in particular, major challenges related to the low abundance of human pathogenic viruses remain, often necessitating costly deep sequencing. Consequently, streamlined wet-laboratory protocols through improvements in sample concentration, nucleic acid extraction, host and background nucleic acid depletion, and possible viral enrichment will be needed to maximize recovery of human infection-relevant viral reads, without substantially increasing sequencing depth. In addition, several analytical limitations related to downstream analysis are also inherent. Long-read sequencing may help overcome some of these by improving genome reconstruction. In parallel, harmonized bioinformatic pipelines, representative reference databases, standardized benchmarking procedures, and transparent reporting practices are needed to improve the comparability and reliability of results. Recent advances in artificial intelligence-assisted analyses offer promising opportunities but require further validation. Finally, clear frameworks for interpreting novel viral signals are also needed to distinguish genuine public health threats from background variation or methodological artifacts.

Addressing these research gaps through coordinated efforts in protocol harmonization, laboratory optimization, and bioinformatic standardization is essential to ensure the reliability of wastewater sequencing as an actionable tool for public health surveillance. This will support its integration into routine infectious disease monitoring, positioning it as a cornerstone of next-generation population-level pathogen surveillance and pandemic preparedness.

## Acknowledgments

M.D.S. received University of Antwerp BOF Seal of Excellence funding (Project ID 55530), and N.U.H. received University of Antwerp BOF DocPro1 funding (Project ID 51266). W.L.C. was supported by the Flanders Innovation & Entrepreneurship agency (VLAIO) under grant number HBC.2024.0273.

## Author contributions

Conceptualization, M.D.S., W.L.C., and P.D.; investigation, M.D.S.; writing – original draft preparation, M.D.S.; writing – review and editing, M.D.S., W.L.C., N.U.H., and P.D.; visualization, M.D.S.; supervision, P.D.; project administration, P.D. All authors have agreed to the published version of the manuscript.

## Declaration of interests

The authors declare no competing interests.

## Declaration of generative AI and AI-assisted technologies in the writing process

During the preparation of this work, the authors used Microsoft Copilot to assist with language editing and grammar. After using this tool/service, the authors reviewed and edited the content as needed and take full responsibility for the content of the published article.
